# Supplementary material for: Direct observation of tunable thermal conductance at solid/porous crystalline solid interfaces induced by water adsorbates
Source: Nat Commun. 2024 Mar 14;15:2304. doi: 10.1038/s41467-024-46473-8 (PMC11258301; doi:10.1038/s41467-024-46473-8)
Supplement: Supplementary file 1 — Supplementary Information [file 41467_2024_46473_MOESM1_ESM.pdf]

Supplementary information

Direct Observation of Tunable Thermal Conductance at Solid/porous Crystalline Solid Interfaces Induced by Water Absorbents

Guang Wang[1#], Hongzhao Fan[1#], Jiawang Li[1], Zhigang Li[1] and Yanguang Zhou[1]*

[1]*Department of Mechanical and Aerospace Engineering, The Hong Kong University of Science and Technology, Clear Water Bay, Kowloon, Hong Kong SAR*

Contents

Supplementary Note 1. The transducer **P2**

Supplementary Note 2. Sensitivity analysis **P6**

Supplementary Note 3. FDTR measurements **P8**

Supplementary Note 4. Molecular dynamics simulations **P12**

Supplementary Note 5. The generalizability of the proposed strategy **P16**

[#] These authors contribute equally. [*]Author to whom all correspondence should be addressed. Email: maeygzhou@ust.hk

Supplementary Note 1. The transducer

S 1.1 Thickness

The thickness of the Au transducer needs to be determined before the frequency-domain thermoreflectance (FDTR) measurements. Here, we sputter the Au on a reference glass slice and measure its corresponding thickness. The glass slide was partially covered by a P.I. tape to form a sharp step, which could be easily peeled off after the sputtering. The thickness of the step was then measured using atomic force microscopy (AFM) (**Figure S1a)**. The average thickness of Au film in the region marked by the red dashed box was around 106 nm (**Figure S1b)**. To make sure the thickness on the reference glass slice is the same as the Au layer on HKUST-1 crystals, we get a cross-section of this sample using the focused ion beam-scanning microscope electron (FIB-SEM). The TEM analysis software DigitalMicrography is used to measure the thickness of the Au film of the Au/HKUST-1 sample (**Figure S2**). The thicknesses of Au film at three different locations are 102.95 nm, 104.88 nm, and 105.17 nm, respectively. The mean thickness of this sample is regarded as ~104.3nm, which is similar to the thickness (i.e., ~106 nm) of Au film on the reference glass slice as obtained by AFM. Therefore, we can think they have the same thickness with acceptable error. Besides, we also include an uncertainty of ~5% of the thickness in the FDTR fitting to consider the error caused by the slight variation of Au thickness.

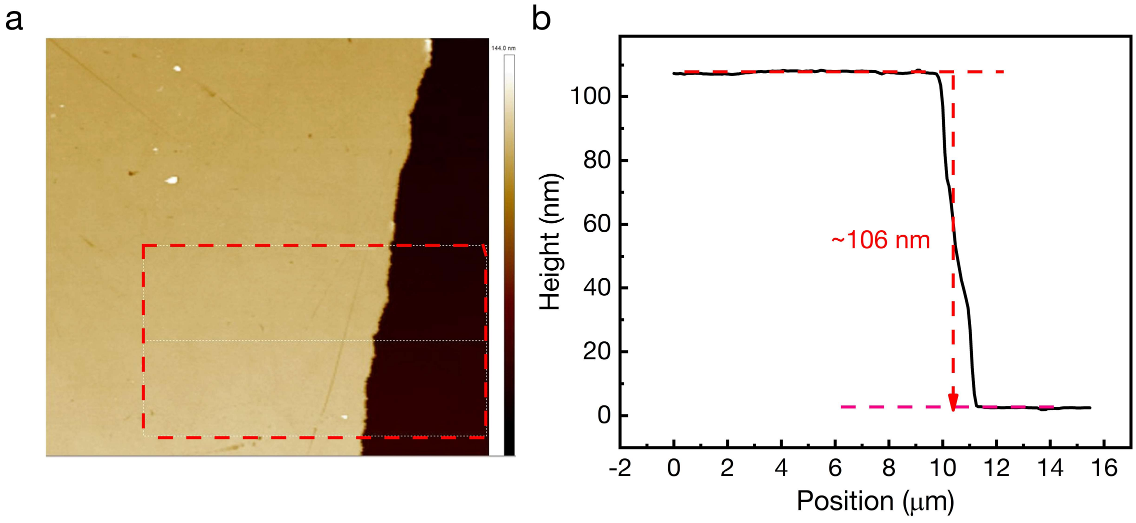

Figure S1 The AFM characterization of Au transducer. (a) The large area scanned across the step of Au film sputtered on a glass slide. (b) The thickness profile of the region marked by the red dashed box in (a).

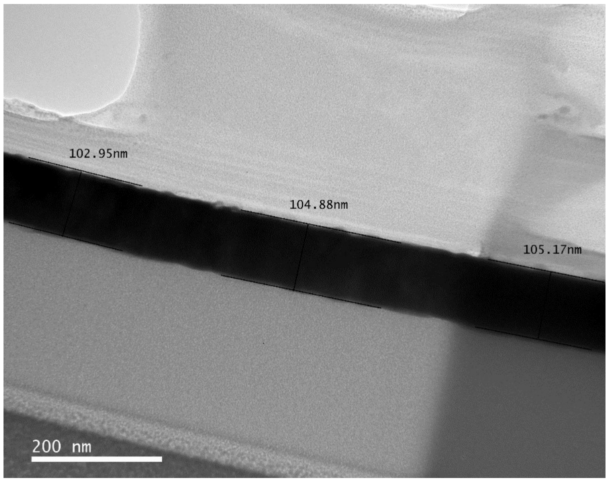

Figure S2 The TEM image of a cross-section of a typical Au/HKUST-1 sample which is prepared by FIB-SEM.

S 1.2 The sample choosing criteria

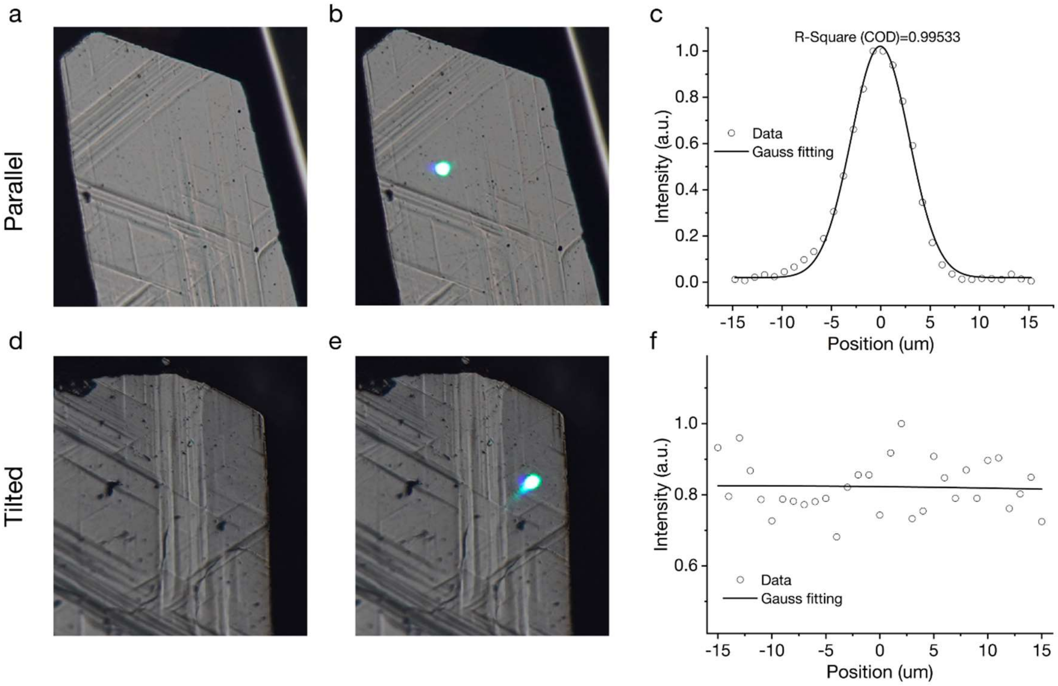

Figure S3 The laser profiles on the samples with parallel and tilted surfaces with respect to the Si substrate. (a) The sample with a parallel surface to the Si substrate and (b) its corresponding laser spot under the microscope. (c) The Gaussian beam profile on the samples with parallel surfaces to the Si substrate. (d) The sample with a tilted surface to the Si substrate and e, its corresponding laser spot under the microscope. (f) The Gaussian beam profile on the samples with tilted surfaces to the Si substrate.

As the size of HKUST-1 crystals in our samples is small and ranges from several tens to several hundreds of μm. Meanwhile, the surface of HKUST-1 crystals on the substrate is different. In our study, we can only measure the thermal transport properties of samples with HKUST-1 crystals parallel to the substrate. Here, the pump laser profile is used to confirm that the surface of HKUST-1 crystals is parallel to the substrate. The laser profile under the optical microscope differs for the HKUST-1 samples with various surfaces. We use a beam offset method to obtain the laser profile. For instance, the laser profile on an HKUST-1 sample with a surface parallel (**Figure S3a**) to the substrate is circular (**Figure S3b**), whereas on the HKUST-1 sample with a surface not parallel to the substrate (**Figure S3d**) has a conical shape (**Figure S3e**). Furthermore, because of the octahedral structure of HKUST-1 crystals, the HKUST-1 surface needs to be parallel to the Si substrate to achieve a uniform sputtered Au film. The laser profile on HKUST-1 parallel surfaces is fitted using the Gaussian function, and the derived laser spot radius is ~3.6 μm (**Figure S3c**), whereas the laser profile on HKUST-1 tilted surfaces cannot be fitted using the Gaussian function (**Figure S3f**). To ensure the accuracy of our measurements, we measure these HKUST-1 samples with relatively large grain sizes (i.e., several hundreds of μm), as shown in **Figure 1a**. Therefore, the grain size of our chosen HKUST-1 samples is believed to be large enough for sputtering a thin Au film, and is much larger than the transducer thickness, which enables us to find an appropriate region for the FDTR measurements.

S 1.3 The thermal conductivity of the Au transducer

As the thickness of the Au transducer in our samples is around 100 nm, the thermal conductivity of the Au transducer should be different from its bulk value. We sputter the Au on a reference fused silica substrate with a size of 1cm × 1cm, which was placed near the HKUST-1 sample during sputtering. We first measured the electrical conductivity of the Au transducer using a 4-probe station. The thermal conductivity of the Au transducer is then determined by the Wiedemann-Franz (W.F.) law: $\kappa_e = L\sigma T$, where L is the Lorentz number (i.e., $2.44 \times 10^{-8}\ \mathrm{W\Omega K^{-2}}$), σ is the electrical conductivity of materials and T is the temperature. It is known that electrons are the main heat carriers in Au, and therefore the thermal conductivity is mainly contributed by electrons. As a result, the measured thermal conductivity using the W.F. law (i.e., 183 W/mK) agrees well with that of our FDTR measurements (**Figure S4**). The thermal conductivity of the Au transducer used in our fitting diffusion model is therefore 183 W/mK.

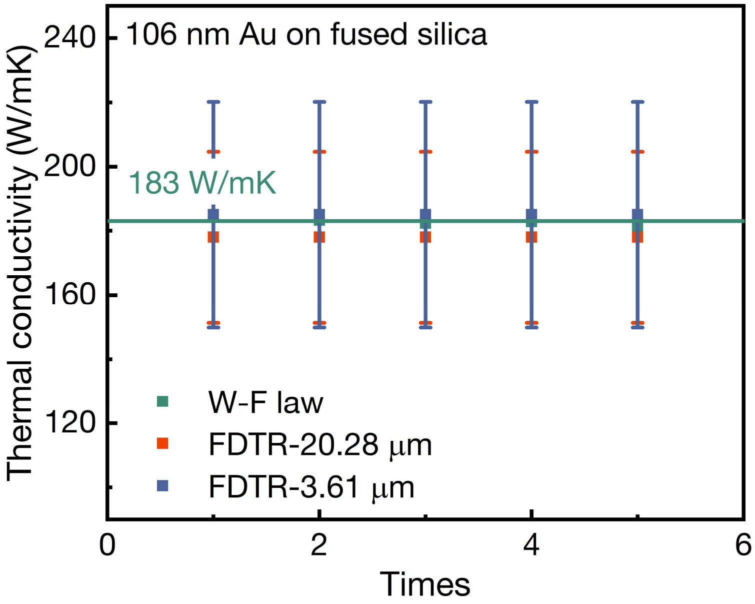

Figure S4 Thermal conductivity measured based on the W.F. law and FDTR with various pump laser spots. The thermal conductivity from the W.F. law is ~183 W/mK, which agrees well with the value from FDTR measurements.

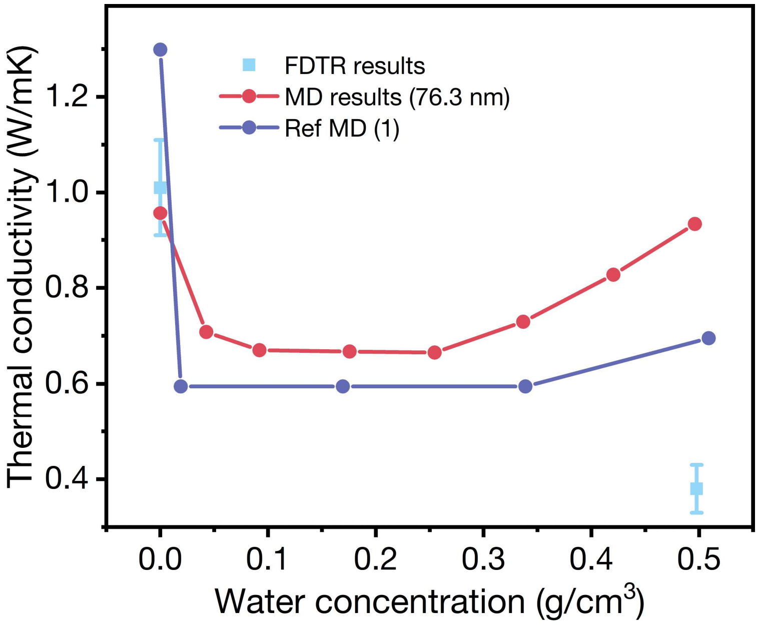

Figure S5 The thermal conductivity of HKUST-1 crystals with various adsorbed water molecules. The experimental results were obtained from FDTR measurements. The molecular dynamics (M.D.) results were calculated by the NEMD simulations. The length of the system in our NEMD simulations is 76.3 nm. The MD results from Ref. [1] were also plotted here for comparison.

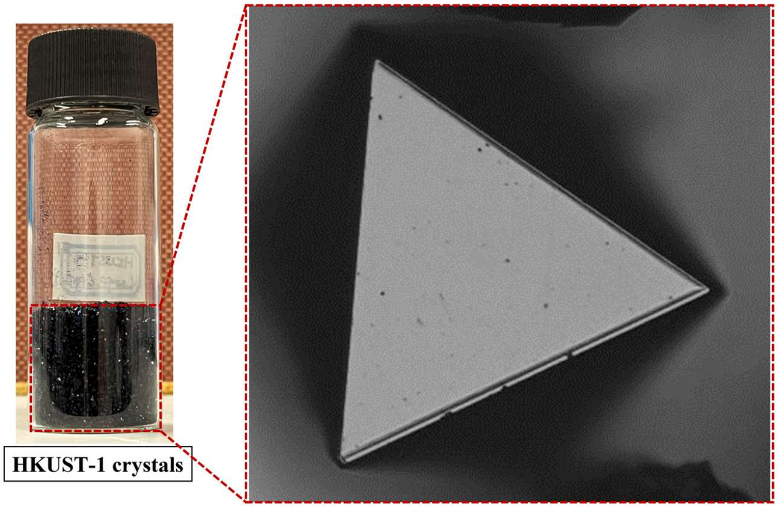

Figure S6 The digital photo of the as-prepared HKUST-1 crystals and the optical FDTR microscope of the Au-coated HKUST-1 crystals. The optical image indicated a smooth and flat surface of our synthesized HKUST-1 crystals.

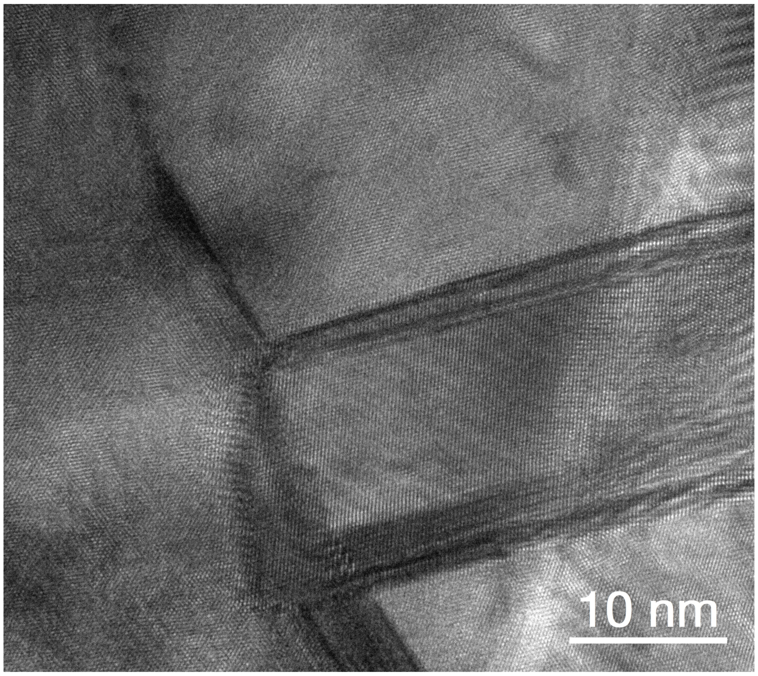

Figure S7 The TEM of the Au layer in our samples. The TEM image indicated that the Au layer in our samples is not a single crystal.

Supplementary Note 2. Sensitivity analysis

The sensitivity of each parameter included in our thermal diffusion model is critical for the fitting of our measured data. If the sensitivity of a specific parameter in the thermal diffusion

model is too low, it is then hardly fitted. The sensitivity of a parameter x, $S(\omega)$, to the measurement can be described as

$$S(\omega) = \frac{\partial \phi(\omega)}{\partial \ln x} \tag{1}$$

where $\phi(\omega)$ is the phase signal. Here, sensitivity analysis of the concerned properties (i.e., the heat capacity of HKUST-1, the thermal conductivity of HKUST-1, and the ITC between HKUST-1 and Au) is performed to ensure the accuracy of our FDTR measurements. While multiple unknown properties can be determined from one FDTR test, the correlation between the sensitivities of these unknown properties will influence the accuracy of the fitting. The material properties for sensitivity analysis and model fitting are shown in **Table S1.**

Table S1 Material properties that are needed for the model fitting at 300 K.

Materials	C (10^6 J $m^{-3}K^{-1}$)	κ ($Wm^{-1}K^{-1}$)	*d* (nm)	ITC ($MWm^{-2}K^{-1}$)
Au	2.56	183*	106**	N.A.
HKUST-1 (Activated)	0.682[1]	To be determined	Semi-infinite	To be determined
HKUST-1 (Saturated)	3.618[1]	To be determined	Semi-infinite	To be determined

*Obtained from the W.F. law. **obtained from AFM measurements.

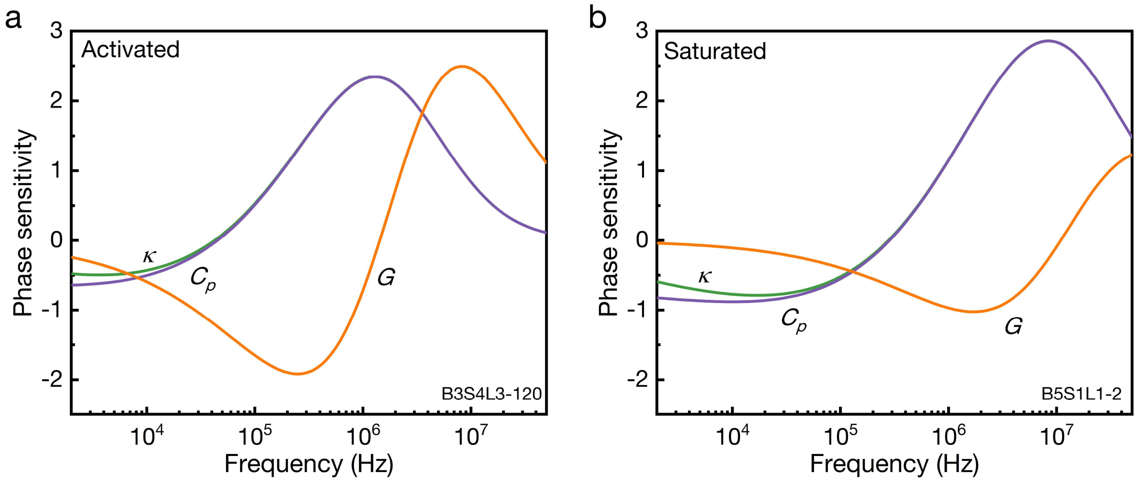

Figure S8 The phase sensitivity analysis of typical samples. The calculated phase sensitivity of C_p, κ of HKUST-1, and ITC of the Au/HKUST-1 heterointerface (a) with and (b) without considering adsorbed water.

The pump radius is measured by the beam offset method[2] with a constant probe radius of 5 μm. The sensitivity analysis for the heat capacity C_p, thermal conductivity (κ) of HKUST-1, and ITC of Au/HKUST-1 heterointerfaces are shown in **Figure S8**. For activated samples and samples with saturated water, the phase sensitivity of κ and C_p is closely correlated, whereas the ITC between Au and HKUST-1 varies differently. Therefore, κ (C_p) of HKUST-1 and ITC of the Au/HKUST-1 heterointerface can be determined from one measurement. In our measurements, the C_p of activated HKUST-1 and HKUST-1 with saturated water taken from Ref.[1] is used as the input, and the κ of HKUST-1 and the ITC of the Au/HKUST-1 heterointerface are then extracted at the same time.

Supplementary Note 3. FDTR measurements

S 3.1 The temperature rise effect on the water adsorbed in HKUST-1

The FDTR uses the pump laser as the heat source, and the temperature rise can be detected by the probe laser. Two continuous-wave (cw) lasers are used in our system. The pump uses a 365 mW (nominapower) diode laser with a wavelength of 445 nm (OBIS 445-365C) which can be modulated bythe lock-in amplifier. The probe is a 20 mW diode laser with a wavelength of 532 nm (OBIS LS532-20). Whereas, if the temperature is too high, the water collected by the HKUST-1 might be evaporated, which may affect the measurements. To minimize the temperature rise caused by the heat source, we use a filter to reduce the power of the applied heat source to 10% of the original value. The heating power on the sample surface is around 2.7 mW after a 27 mW original laser pump source goes through the filter. This heating power can generate a clear thermoreflectance signal in our FDTR apparatus but will not evaporate the water, as described in the following.

The temperature rise in FDTR measurements can be estimated based on the radially symmetric heat diffusion equation[3]. For a semi-infinite solid, the frequency domain solution for a surface heated by a unit power at angular frequency ω is

$$g(r) = \frac{exp(-qr)}{2\pi\kappa r} \tag{2}$$

and

$$q^2 = \frac{i\omega}{D} \tag{3}$$

where κ is the thermal conductivity of the solid, D is the thermal diffusivity, and r is the radial coordinate. We can then apply the Hankel transform[4] to $g(r)$ for the convolution of $g(r)$ with laser intensities in a co-aligned pump and probe system. The Hankel transform of $g(r)$ is

$$G(k) = 2\pi\int_0^\infty g(r)J_0(2\pi kr)rdr = \frac{1}{\kappa\sqrt{(4\pi^2k^2+q^2)^{1/2}}} \tag{4}$$

where the J_0 is the zeroth-order Bessel function of the first kind. If the surface is heated by a pump laser beam with a Gaussian distribution intensity $p(r)$, which has a form of

$$p(r) = \frac{2A}{\pi w_0^2} exp\left(\frac{-2r^2}{w_0^2}\right) \tag{5}$$

in which, A is the amplitude of the adsorbed heat power, w_0 is the $1/e^2$ radius of the pump. The laser intensity in the frequency domain can be obtained using the Hankel transform

$$P(k) = A\, exp\left(\frac{-\pi^2k^2w_0^2}{2}\right) \tag{6}$$

The temperature distribution at the surface is therefore expressed as the inverse transform of the product of $G(k)$ and $P(k)$

$$\theta(r) = 2\pi\int_0^\infty P(k)G(k)J_0(2\pi kr)kdk \tag{7}$$

In our FDTR apparatus, the probe laser detects the temperature rise based on the change of surface reflectivity. Considering the probe laser intensity also has a Gaussian distribution, the weighted average temperature distribution acquired by a probe beam with a radius of w_1 is

$$\Delta T = \frac{4}{w_1^2}\int_0^\infty \theta(r) exp\left(\frac{-2r^2}{w_1^2}\right) rdr \tag{8}$$

We use the Matlab script developed by Braun *et al.*[5] based on Eq. (8) to calculate the temperature rise of our systems caused by the applied heat source. The temperature rise results from two parts: 1, a steady-state response resulting from the average adsorbed power, and 2, a modulated response from oscillations at the modulation frequency about the average power. It is widely accepted that the steady-state temperature rise dominates the global temperature rise[6].

In our calculations, a two-layer model is used to represent our Au/HKUST-1 systems. The Au layer has a thickness of 105 nm, a heat capacity of 2.56 MJ/m^3K, and an isotropic thermal conductivity of 183 W/mK. The saturated HKUST-1 layer is assumed to be a semi-infinite layer, which has a heat capacity of 3.618 MJ/m^3K and an isotropic thermal conductivity of 0.416 W/mK. The interfacial thermal conductance (ITC) between the Au layer and the saturated HKUST-1 layer is set as 21.66 MW/m^2K (i.e., the mean value of our experimental results). Our results show that the largest temperature rise of the laser spot is ~9 K and ~1.0 K when the modulation frequency is 2000 Hz (**Figure S9a**) and 10 MHz (**Figure S9b**), respectively. In our calculations, we have considered that only 20% of a 532 nm wavelength laser is absorbed by the Au transducer[7]. The temperature rise for the low modulation frequency

is higher than that for the high modulation frequency, which agrees with other Ref.[3]. As suggested by Jiang *et al.*[8] the temperature rise caused by the applied heat source should not exceed 10 K or 10% of the absolute temperature. Therefore, the temperature rise in our FDTR measurements is believed to be acceptable. Meanwhile, it is known that the thermal conductivity of the Au transducer is much higher than that of HKUST-1, which makes the temperature rise caused by the applied heat source in MOFs much smaller than the above-calculated values.

Our FDTR measurements are conducted at ~298 K, and the maximum temperature in the samples can be regarded as ~307 K, considering the temperature rise caused by the applied heat source. Our thermal gravimetric analysis (TGA) shows that the weight loss ratio of the saturated HKUST-1 at 307 K is 4.6% (**Figure S10a**). The modulation frequency of the pump laser in FDTR measurements ranges from 2000 Hz to 50 MHz, which indicates that the maximum temperature rise caused by the applied heat source should be much lower than 9 K. The maximum weight loss ratio of our samples caused by the applied heat source in all our FDTR measurements should be much smaller than 4.6%. As a result, the applied heat source has little influence on our measured thermal properties. Furthermore, we swept 5 times at the same location using the same pump laser and found that the measured phase lag curves were almost the same (**Figure S10b**). This once again indicates that the applied heat source has little influence on our measured results.

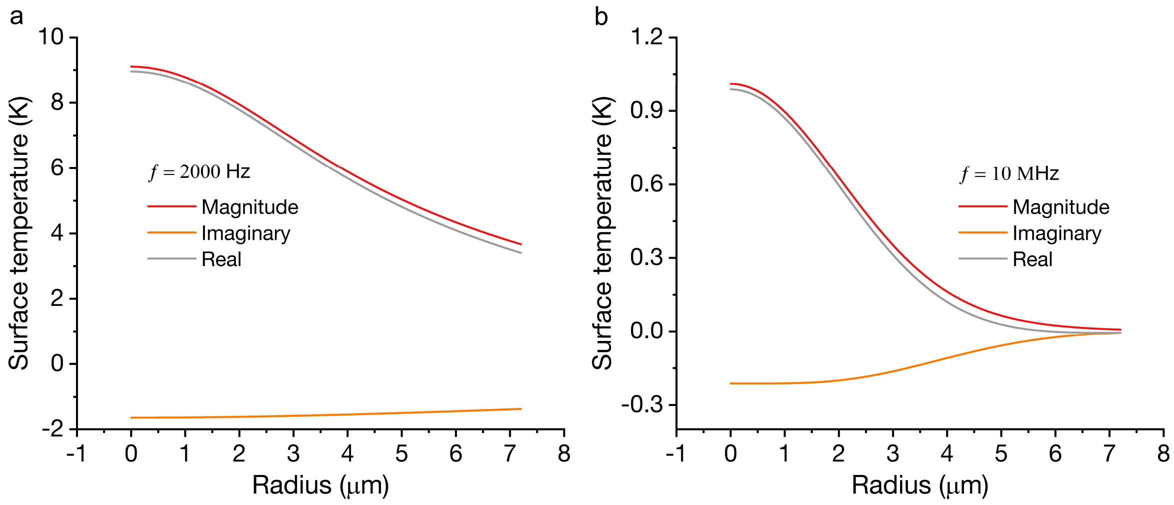

Figure S9 The temperature rise as a function of radius on the Au transducer layer at modulation frequencies of (a) 2000 Hz and (b) 10 MHz.

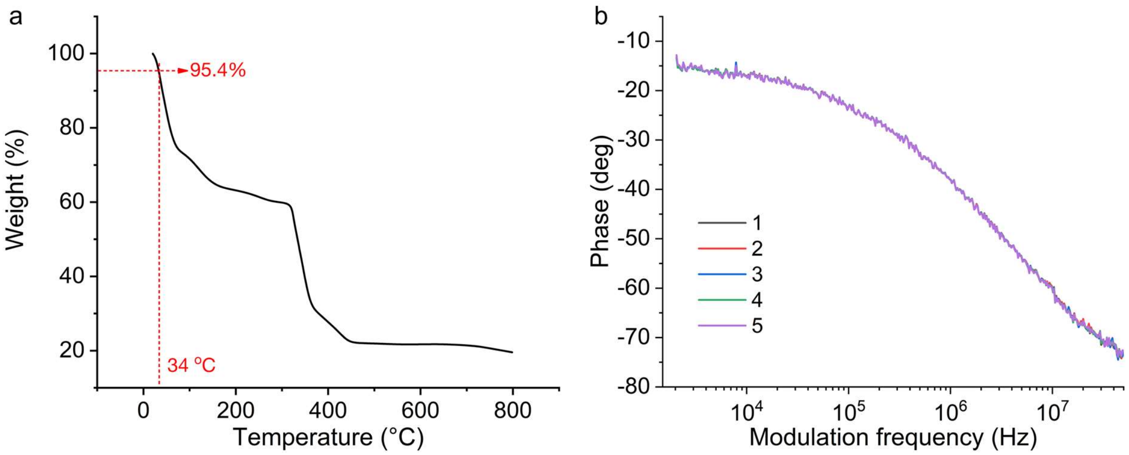

Figure S10 (a) The TGA test of saturated HKUST-1 with a ramp rate of 10K/min. (b) The phase lag of one typical saturated sample which was swept 5 times at the same position.

S 3.2 The experimental results of ITC

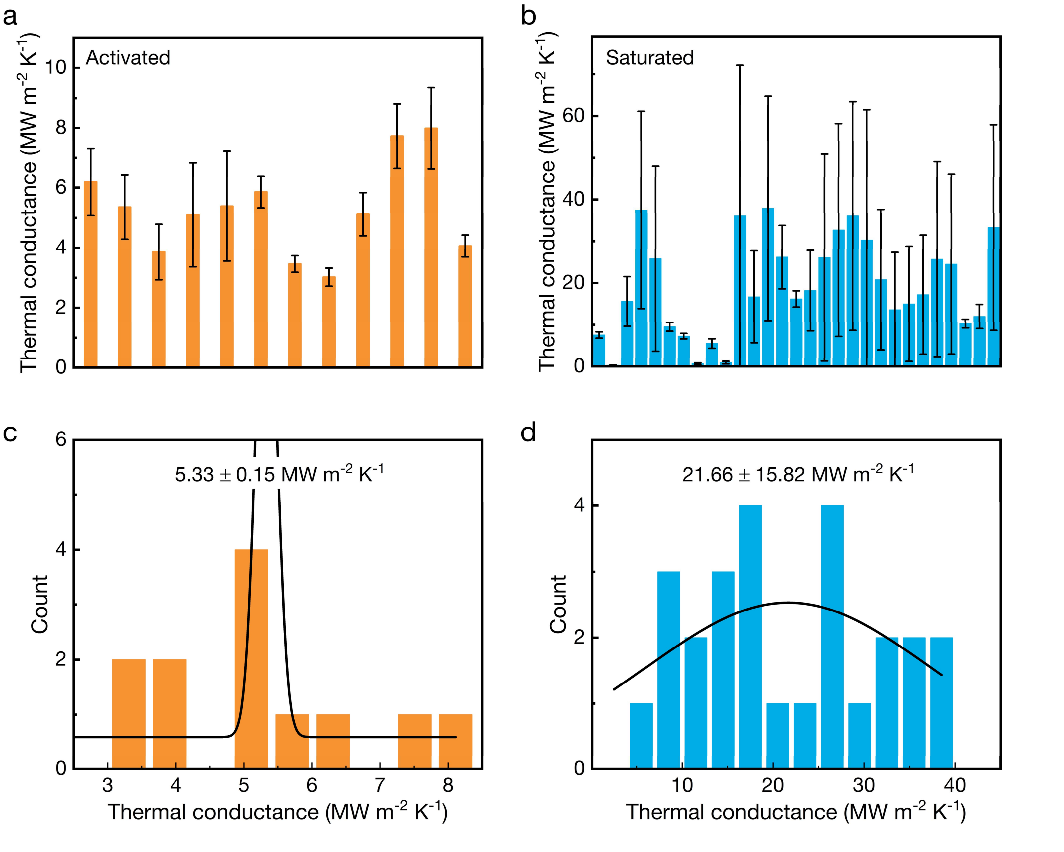

Figure S11 Experimental measurements. (a) The thermal conductance of activated and (b) saturated Au/HKUST-1 heterointerfaces, and the corresponding Gaussian fitting for (c) activated and (d) saturated samples.

Over one hundred samples from six batches in total are chosen for measuring the thermal conductivity of HKUST-1 and ITC of Au/HKUST-1 heterointerfaces to reduce the uncertainty. The uncertainties in our measured results are resulted from the uncertainties in all material properties used for fitting. The results with uncertainties larger than 100% are excluded.

Figure S11 shows the measured ITCs for both activated and saturated samples. The average ITCs of both activated and saturated samples were obtained via a Gaussian fitting. The data sets yielded average ITCs of 5.33 MW/m^{2}K and 21.66 MW/m^{2}K for activated and saturated Au/HKUST-1 heterointerfaces, respectively.

Supplementary Note 4. Molecular dynamics simulations

S 4.1 Water molecules adsorption

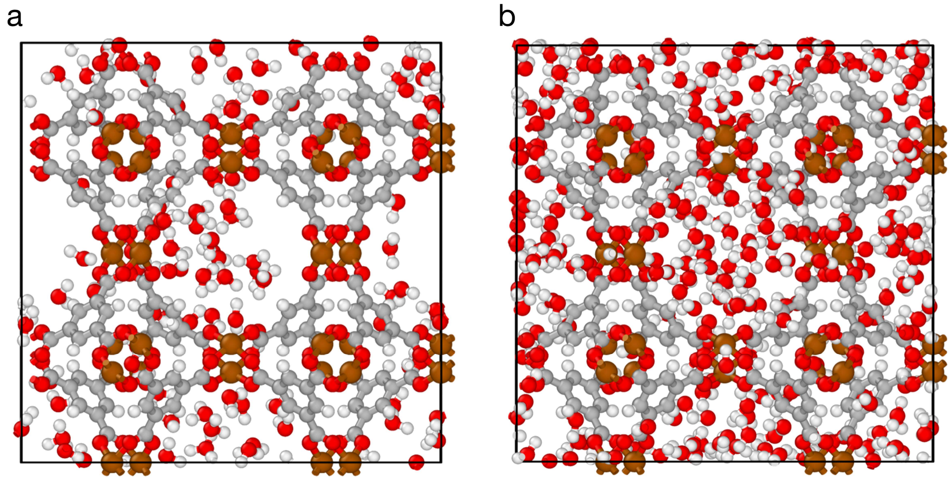

Figure S12 The models constructed by GCMC simulations. The HKUST-1 with water densities of (a) 0.16 g/cm^3 and (b) 0.41 g/cm^3. The hydrogen atoms, oxygen atoms, carbon atoms, and copper atoms are represented by white, red, grey, and brown balls, respectively.

The water adsorption process of HKUST-1 is simulated by performing the Grand canonical Monte Carlo (GCMC) simulations. The GCMC simulations are implemented by the LAMMPS software[9]. All GCMC simulations are performed at 300K. During the process of water adsorption, the GCMC simulation is invoked every 500 timesteps. On these timesteps, around 500 GCMC exchanges are attempted. The average Monte Carlo (MC) moves approximately equal to the number of water molecules in the simulation box. For the GCMC exchanges, water molecules can be inserted into or deleted from the simulation box. The probabilities of deletions and insertions are equal. For each MC move, the move of water molecules can be a translation and a rotation. The chemical potential of GCMC simulations is considered by setting the

pressure as 1 bar. The atomic configuration is output during the GCMC simulations to obtain models containing various water molecules. The models containing different water molecules are displayed in **Figure S12**.

S 4.2 Nonequilibrium molecular dynamics simulations

In nonequilibrium molecular dynamics (NEMD) simulations, a symmetrical model was adopted to calculate the ITC. The model and the settings of NEMD simulations are depicted in **Figure S13**. In all our NEMD simulations, the thermal energy flows from the heat source to the heat sink and crosses two interfaces. The ITC is the mean value of the two calculated values at these two interfaces. To reduce the uncertainty, all our calculated ITC is averaged by 3 different simulations.

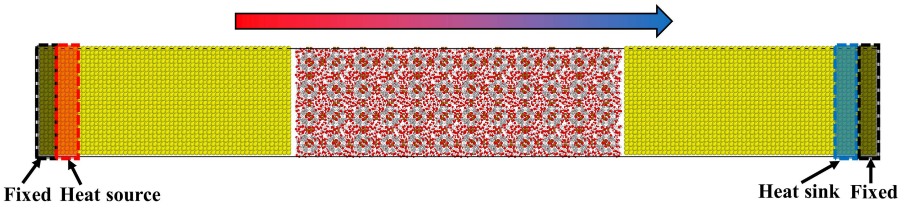

Figure S13 The schematic of the NEMD simulation. The heat flows from the heat source to the heat sink. Two regions of Au atoms at the distal end of the model with a width of 13 Å are fixed in the M.D. simulations. Au atoms in the region with a width of 26 Å are selected for applying source and sink thermostats.

S 4.3 The interfacial interaction

In all our simulations, the interfacial interactions between Au and HKUST-1 are described by the universal force field (UFF)[10]. The ITC of Au/HKUST-1 heterointerfaces described by using UFF potential agrees well with our experimental measurements (**Figure 3** in our main text). While the interactions between Au and water molecules can be described by the UFF force field, the detailed parameters usually need to be adapted as the interaction between Au and water molecules is quite diverse[11–18]. This is because the interaction between Au and water molecules is affected by many factors such as the cleanliness[19] and roughness[11] of Au substrate, which also leads to the measured contact angle of water on Au substrate distributed in a wide range[19,20]. Hence, to obtain the experimental comparable ITC in our NEMD simulations, it is necessary to adjust the interactions between Au and water molecules. In this manuscript, we adjusted the energy parameters in the standard 12/6 Lennard-Jones potential originally proposed by Hu *et al.*[12] to ensure our calculated ITC of Au/HKUST-1 heterointerfaces agree

with our FDTR measurements. Consequently, the strong (weak) interfacial interactions with parameters of $\varepsilon = 0.01279$ eV ($\varepsilon = 0.01039$ eV) and $\sigma = 3.6$ Å ($\sigma = 3.6$ Å) in the adopted UFF potential were used in simulations. The contact angle is 79° (**Figure S14a**) and 110° (**Figure S14b**) for the strong and weak adhesion Au/HKUST-1 heterointerfaces, respectively. Our calculated ITCs for the weak (strong) adhesion Au/HKUST-1 heterointerfaces with saturated water molecules are ~10 M.W./m^{2}K (~35 M.W./m^{2}K), which agrees reasonably with the upper and lower boundaries of our FDTR measurements.

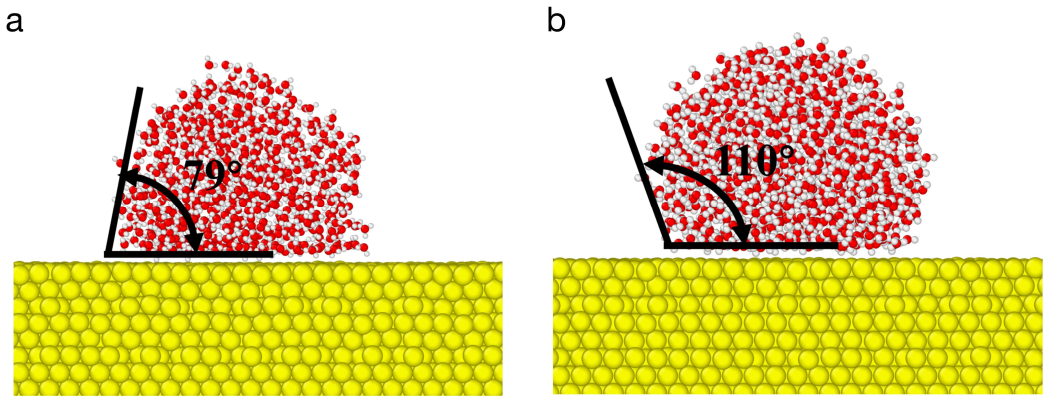

Figure S14 The calculated contact angles. The calculated contact angle of two sets of (a) strong and (b) weak interfacial interaction parameters between Au and water molecules.

S 4.4 The vibrational density of state (VDOS) near the interface

For the interfacial thermal transport, the vibrations near the interface may be different from that in the area far away from the interface. This is because the atoms in the interfacial region are strongly affected by both the two contacting materials. To analyse the impacts of interfacial interaction on atomic vibrations, we defined the interface region and discussed the VDOS spatial variation. Based on the energy distribution along the direction perpendicular to the interface **(Figure S15)**, the interfacial region can be regarded to have a thickness of ~0.6 nm, which includes three layers of Au atoms. We then calculate the VDOS of Au at various positions with different distances from the interface. Our results show that the VDOS of the first layer of Au near the interface has been changed due to the interface effect, and the VDOS of Au will converge with the distance from the interface **(Figure S15)**. We further find that the HKUST with or without water molecules has little influence on the VDOS of Au atoms near the interface **(Figure S15)**. This is because the interaction among Au atoms is stronger than that between Au and water molecules or HKUST-1. Furthermore, the thickness of the Au layers affected by the interface is ~0.6 nm, which is much smaller than the dimension of our

Au/HKUST-1 samples. Therefore, it should be reasonable to assume that the interfaces in our FDTR fitting models and NEMD calculations have a zero thickness.

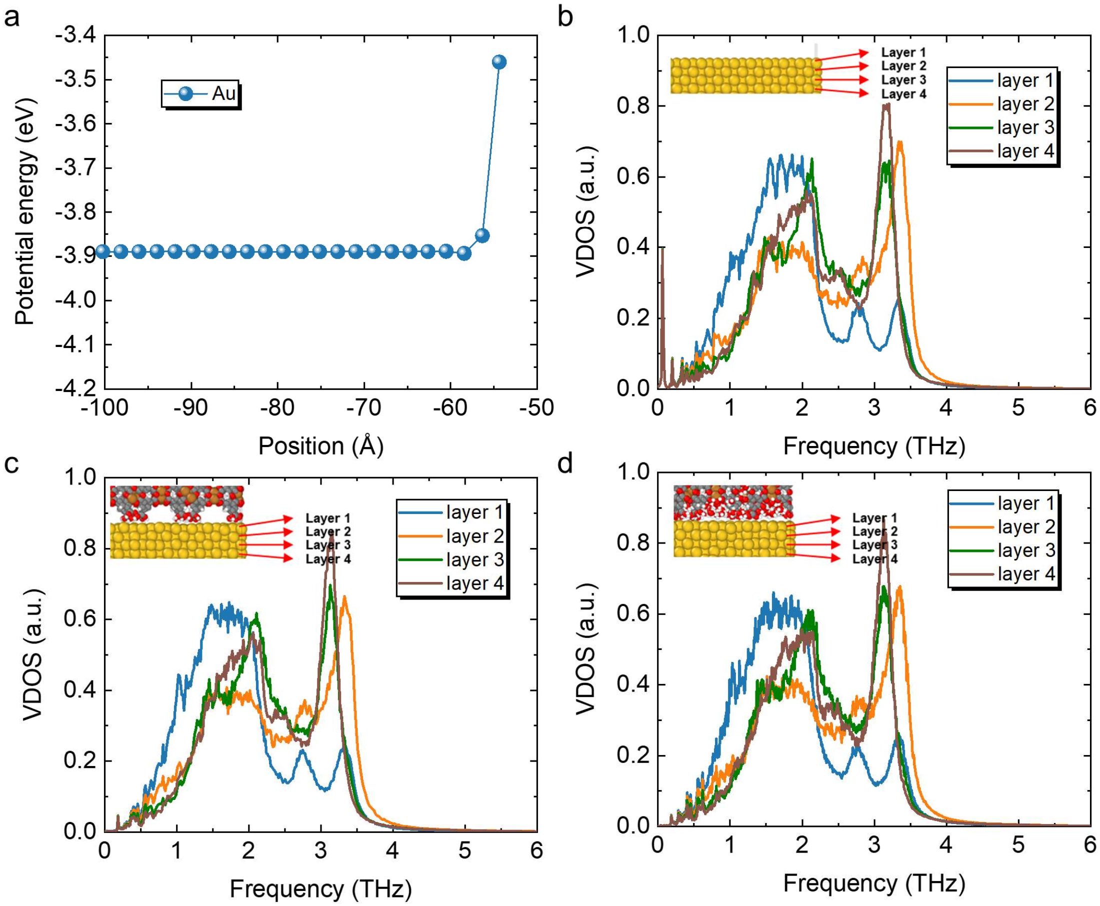

Figure S15 The VDOS near the interface. (a) The potential energy distribution of Au atoms near the interface, in which each point means the averaged potential energy of one layer of Au atoms. (b) The VDOS of Au atoms in different potions at bare Au interface. (c) The VDOS of Au atoms in different potions at Au-HKUST-1 interface with water density of 0 g/cm^3. (d) The VDOS of Au atoms in different potions at Au-HKUST-1 interface with water density of 0.5 g/cm^3.

S 4.5 The contribution of the Au/HKUST-1 channel to ITC

In this paper, the ITC spectrum is characterized by the frequency domain direct decomposition method. It is found that the vibrational transmission coefficient is largely increased when the water molecules are absorbed into the systems (**Figure 4** in our main text). Meanwhile, we also quantify the contribution of the Au/HKUST-1 channel to ITC (**Figure S16**). Our results show that the contribution of the Au/HKUST-1 channel to ITC is almost

unchanged with the adsorbed water molecules. That means that the adsorbed water molecules at the interface will not affect the Au/HKUST channel and will add an additional Au/water channel for thermal energy exchange across the interfaces.

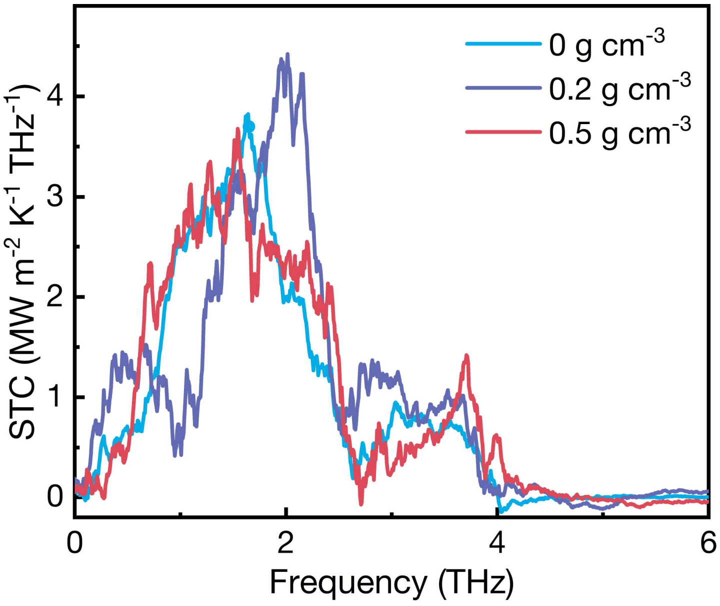

Figure S16 The contribution of the Au/HKUST-1 channel. The ITC spectrum contributed from the Au/HKUST-1 channel with strong Au/water adhesion.

Supplementary Note 5. The generalizability of the proposed strategy

To show the generalizability of our proposed strategy, we further investigated the thermal transport in two other MOF/substrate systems (i.e., UiO-66/Au and MOF-55/Au interfaces) using either experiments or MD simulations. The chosen UiO-66[21,22] and MOF-505[23] have good water adsorption capacity and stability.

S 5.1 The synthesis of Au/UiO-66 samples and the corresponding FDTR measurement

The UiO-66 crystals were prepared by modifying the method reported by Christopher A. Trickett[24]. In detail, a solvent of N, N-diethylformamide (DEF) was firstly dried by molecular sieves for several days. Then, $ZrOCl_2 \cdot 8H_2O$ (0.037 mmol) and H_2BDC were dissolved separately in DEF (1 mL) by ultrasonication for 5 mins. They were then mixed in a glass vial, which can be tightly capped, and the formic acid was added to the mixture to form a white color turbid solution. The solution was placed in the oven (preheated to 135 °C) for 48 hours. Block crystals were then obtained, followed by a washing process using DMF twice a day for

two days. These bright crystals were activated under a vacuum at 60 ºC for two days before use. The SEM images (**Figure S17**) and the PXRD (**Figure S18**) show that the as-prepared UiO-66 possesses good crystallinity, with grain size ranging from several μm to hundreds of μm. It is noted that some peaks were not observed in the measured XRD spectrum compared to the simulated results. This is because the crystals are not ground into fine powders.

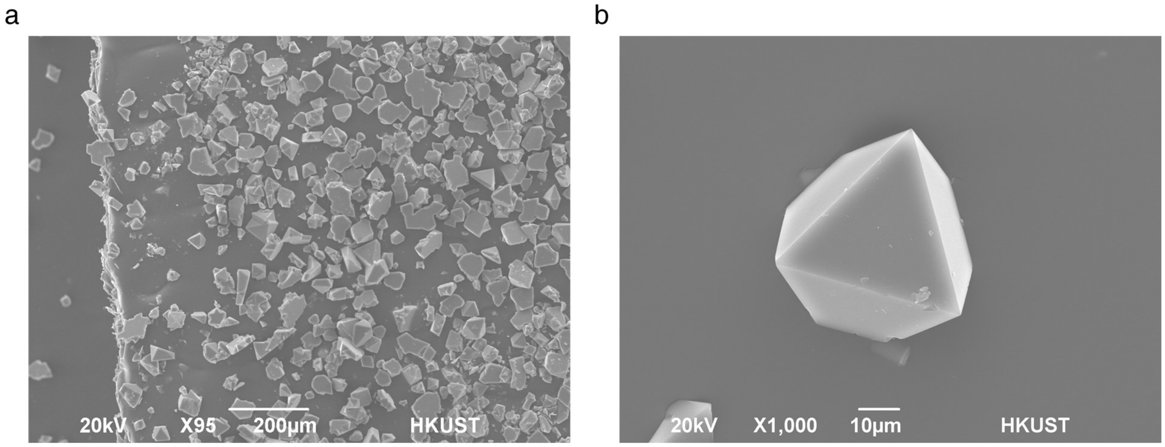

Figure S17 The SEM images of as-prepared UiO-66 crystals under different amplitudes. (a) 95× and (b) 1000×.

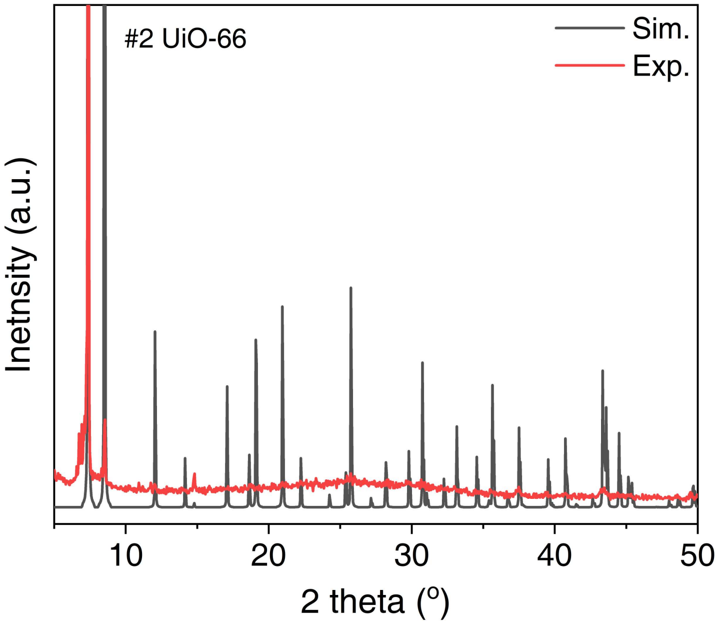

Figure S18 The PXRD patterns of UiO-66 crystals.

We then coated the Au film on the UiO-66 crystals as we did for HKUST-1 crystals, and selected suitable samples for our FDTR measurements. Similarly, the saturated samples were

prepared by immersing the activated UiO-66 crystals in water for ~2 hours. The sensitivity of FDTR measurements for Au/UiO-66 systems is quite similar to that of the Au/HKUTS-1 systems because UiO-66 crystal has a similar thermal conductivity with HKUST-1 crystal (see the results below for details). Following the approach proposed by Babaei *et. al.*[1], the heat capacity of saturated UiO-66 is estimated basing $C_{saturated\ MOF} = C_{activated\ MOF} + \varphi C_{adsorbate}$, in which φ is the void fraction and has a value of 47% for UiO-66 [22]. The calculated $C_{activated\ UiO\text{-}66}$ is 0.88 MJ/m^{3}K based on MD simulations as suggested in Ref.[22]. The heat capacity of water C_{water} is 4.19 MJ/m^{3}K which is directly taken from Ref.[1]. Therefore, the heat capacity of saturated UiO-66 can be calculated and has a value of ~2.9 MJ/m^{3}K. The thermal conductivity of UiO-66 and the ITC between UiO-66 and the Au film can then be obtained (**Figure S19**). Our results show that the thermal conductivity of UiO-66 is reduced from 0.67±0.13 W/mK to 0.23±0.03 W/mK when saturated water is adsorbed (**Figure S20**), and the corresponding ITC between UiO-66 crystals and the Au film increases from 16.4±2.9 MW/m^{2}K to 19.7±3.15 MW/m^{2}K.

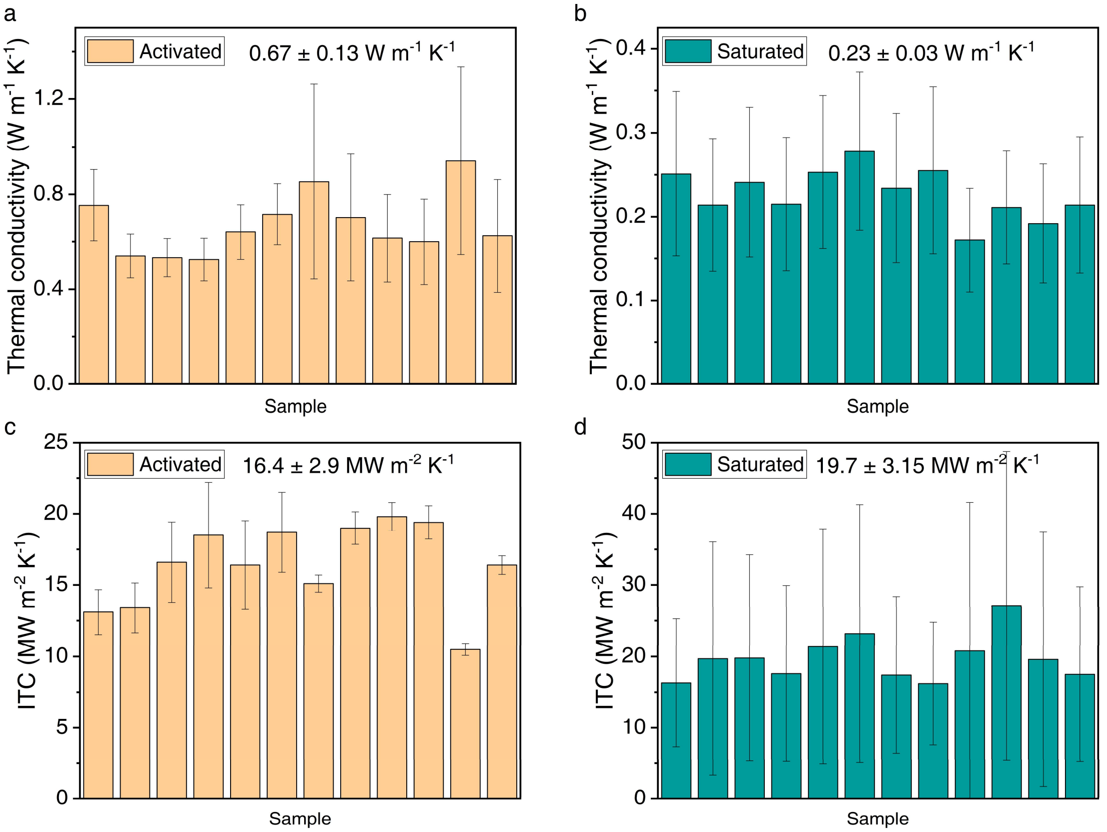

Figure S19 The thermal conductivity of UiO-66 at (a) activated and (b) saturated states. The interfacial thermal conductance between UiO-66 and Au at (c) activated and (d) saturated states.

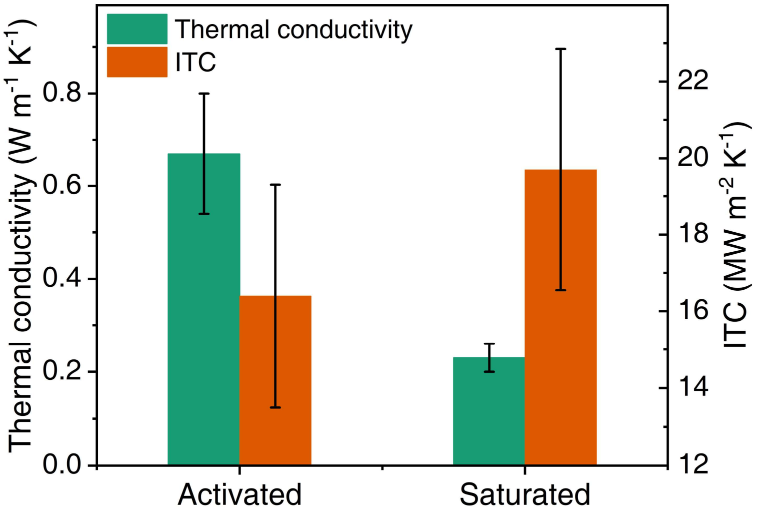

Figure S20 The thermal conductivity of UiO-66 and the interfacial thermal conductance of UiO-66/Au samples with/without adsorbed water molecules.

S 5.2 Thermal transport across MOF-505/Au interfaces

To further verify the enhancement of interfacial thermal transport on other MOFs constructed heterointerfaces, we also performed nonequilibrium molecular dynamics (NEMD) simulations to calculate the interfacial thermal transport between MOF-505 and Au. The model used in NEMD simulations was built in the same way as we did for the Au/HKUST-1 systems, and the size of the system was 5.6 nm × 4.8 nm × 42.6 nm. A forcefield developed based on first-principles calculations was applied to describe the interatomic interactions of the MOF-505 framework[22]. The interaction among Au atoms and water molecules was the same for the Au/HKUST-1 systems with adsorbed water. The weak interaction parameters between Au and a water molecule in the **SI Note 4** were chosen to model the interaction between Au and water. Other interaction parameters are the same as those used in the previous calculations of the Au/HKUST-1 heterointerface. The long-range electrostatic interactions were also considered. The NEMD simulation and water adsorption processes are the same as our previous calculations for HKUST-1/Au systems with/without adsorbed water. Our results show that the ITC of activated Au/MOF-505 heterointerface is 7.72±0.82 MW/m^{2}K, and increases to 19.56±1.81 MW/m^{2}K when saturated water molecules are adsorbed (**Figure S21**).

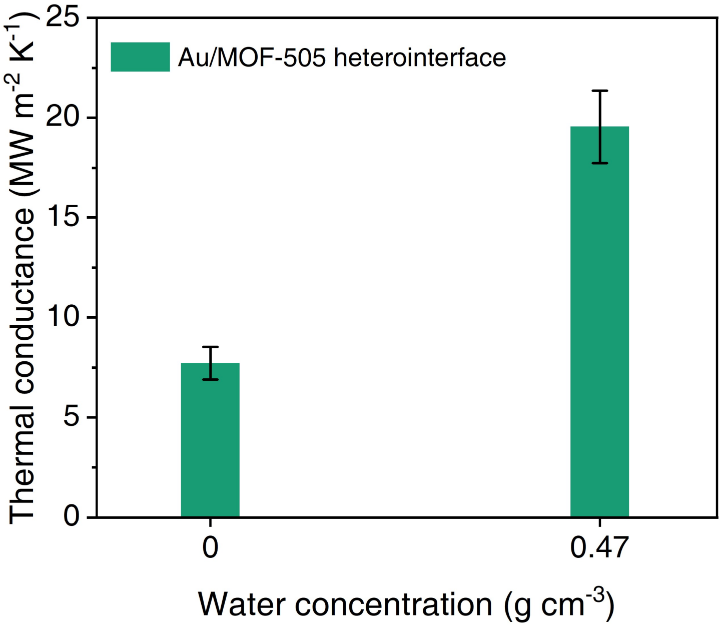

Figure S21 The simulated interfacial thermal conductance of Au/MOF-505 heterointerfaces under different water concentrations.

In summary, the strategy utilizing adsorbed water we proposed here is a general way to enhance the interfacial thermal transport between MOF and substrates, e.g., the maximum enhancement of the ITC is 7.1 times for Au/HKUST-1 interfaces, 1.7 times for Au/UiO-66 interfaces and 3.1 times for Au/MOF-505 interfaces. The enhancement depends on the morphologies and physical properties of MOFs and the corresponding interfaces.

References

1. Babaei, H. *et al.* Observation of reduced thermal conductivity in a metal-organic framework due to the presence of adsorbates. *Nat. Commun.* **11**, 4010 (2020).

2. Feser, J. P. & Cahill, D. G. Probing anisotropic heat transport using time-domain thermoreflectance with offset laser spots. *Rev. Sci. Instrum.* **83**, 104901 (2012).

3. Cahill, D. G. Analysis of heat flow in layered structures for time-domain thermoreflectance. *Rev. Sci. Instrum.* **75**, 5119–5122 (2004).

4. Ohsone, Y., Wu, G., Dryden, J., Zok, F. & Majumdar, A. Optical measurement of thermal contact conductance between wafer-like thin solid samples. *J. Heat Transf.* **121**, 954–963 (1999).

5. Braun, J. L., Szwejkowski, C. J., Giri, A. & Hopkins, P. E. On the steady-state temperature rise during laser heating of multilayer thin films in optical pump–probe techniques. *J. Heat Transf.* **140**, (2018).

6. Braun, J. L. & Hopkins, P. E. Upper limit to the thermal penetration depth during modulated heating of multilayer thin films with pulsed and continuous wave lasers: A numerical study. *J. Appl. Phys.* **121**, 175107 (2017).

7. Yang, J., Maragliano, C. & Schmidt, A. J. Thermal property microscopy with frequency domain thermoreflectance. *Rev. Sci. Instrum.* **84**, 104904 (2013).

8. Jiang, P. & Ban, H. Transient and steady-state temperature rise in three-dimensional anisotropic layered structures in pump-probe thermoreflectance experiments. *J. Phys. Appl. Phys.* **54**, 035304 (2020).

9. Plimpton, S. Fast Parallel Algorithms for short-range molecular dynamics. *J. Comput. Phys.* **117**, 1–19 (1995).

10. Rappe, A. K., Casewit, C. J., Colwell, K. S., Goddard, W. A. I. & Skiff, W. M. UFF, a full periodic table force field for molecular mechanics and molecular dynamics simulations. *J. Am. Chem. Soc.* **114**, 10024–10035 (1992).

11. Wu, C.-D., Kuo, L.-M., Lin, S.-J., Fang, T.-H. & Hsieh, S.-F. Effects of temperature, size of water droplets, and surface roughness on nanowetting properties investigated using molecular dynamics simulation. *Comput. Mater. Sci.* **53**, 25–30 (2012).

12. Hu, H. & Sun, Y. Effect of nanopatterns on Kapitza resistance at a water-gold interface during boiling: A molecular dynamics study. *J. Appl. Phys.* **112**, 053508 (2012).

13. Hautman, J., Halley, J. W. & Rhee, Y.-J. Molecular dynamics simulation of water beween two ideal classical metal walls. *J. Chem. Phys.* **91**, 467 (1998).

14. Ju, S.-P. A molecular dynamics simulation of the adsorption of water molecules surrounding an Au nanoparticle. *J. Chem. Phys.* **122**, 094718 (2005).

15. Aksoy, M. M., AlHosani, M. & Bayazitoglu, Y. Thermal resistance for Au–water and Ag–water interfaces: molecular dynamics simulations. *Int. J. Thermophys.* **42**, 87 (2021).

16. Ju, S.-P. & Chang, J.-G. A molecular dynamics simulation investigation into the behavior of water molecules inside Au nanotubes of various sizes. *Microporous Mesoporous Mater.* **75**, 81–87 (2004).

17. Hu, H. & Sun, Y. Molecular dynamics simulations of disjoining pressure effect in ultra-thin water film on a metal surface. *Appl. Phys. Lett.* **103**, 263110 (2013).

18. Huang, D., Ma, R., Zhang, T. & Luo, T. Origin of hydrophilic surface functionalization-induced thermal conductance enhancement across solid–water Interfaces. *ACS Appl. Mater. Interfaces* **10**, 28159–28165 (2018).

19. Smith, T. The hydrophilic nature of a clean gold surface. *J. Colloid Interface Sci.* **75**, 51–55 (1980).

20. Rafiee, J. *et al.* Wetting transparency of graphene. *Nat. Mater.* **11**, 217–222 (2012).

21. Cavka, J. H. *et al.* A new zirconium inorganic building brick forming metal organic frameworks with exceptional stability. *J. Am. Chem. Soc.* **130**, 13850–13851 (2008).

22. Wieme, J. *et al.* Thermal engineering of metal–organic frameworks for adsorption applications: a molecular simulation perspective. *ACS Appl. Mater. Interfaces* **11**, 38697–38707 (2019).

23. Qi, Z.-P., Yang, J.-M., Kang, Y.-S., Guo, F. & Sun, W.-Y. Facile water-stability evaluation of metal–organic frameworks and the property of selective removal of dyes from aqueous solution. *Dalton Trans.* **45**, 8753–8759 (2016).

24. Trickett, C. A. *et al.* Definitive molecular level characterization of defects in UiO-66 crystals. *Angew. Chem. Int. Ed.* **54**, 11162–11167 (2015).
