# Supplementary material for: Direct observation of tunable thermal conductance at solid/porous crystalline solid interfaces induced by water adsorbates
Source: Nat Commun. 2024 Mar 14;15:2304. doi: 10.1038/s41467-024-46473-8 (PMC11258301; doi:10.1038/s41467-024-46473-8)
Supplement: Supplementary file 4 — Lasing Reporting Summary [file 41467_2024_46473_MOESM4_ESM.pdf]

Corresponding author(s): Yanguang Zhou

Lasing Reporting Summary

Nature Research wishes to improve the reproducibility of the work that we publish. This form is intended for publication with all accepted papers reporting claims of lasing and provides structure for consistency and transparency in reporting. Some list items might not apply to an individual manuscript, but all fields must be completed for clarity.

For further information on Nature Research policies, including our data availability policy, see Authors & Referees.

• Experimental design

Please check: are the following details reported in the manuscript?

1. Threshold

Plots of device output power versus pump power over a wide range of values indicating a clear threshold

- [ ] Yes
- [x] No

The laser power is not crucial for our experiment and we conduct the experiment under a stable power range.

2. Linewidth narrowing

Plots of spectral power density for the emission at pump powers below, around, and above the lasing threshold, indicating a clear linewidth narrowing at threshold

- [ ] Yes
- [x] No

The experiments are conducted under a stable power range. The linewidth narrowing would not affect our experiment.

Resolution of the spectrometer used to make spectral measurements

- [ ] Yes
- [x] No

The spectrometer is not used in our experiments.

3. Coherent emission

Measurements of the coherence and/or polarization of the emission

- [ ] Yes
- [x] No

The coherence and polarization of the emission is insensitive to our FDTR measurement, thus we did not measure it.

4. Beam spatial profile

Image and/or measurement of the spatial shape and profile of the emission, showing a well-defined beam above threshold

- [x] Yes
- [ ] No

In Supplementary Information Figure S3.

5. Operating conditions

Description of the laser and pumping conditions
Continuous-wave, pulsed, temperature of operation

- [x] Yes
- [ ] No

In Supplementary Information Note 3, we described wavelength and the power of the laser we used and the temperature of operation.

Threshold values provided as density values (e.g. W cm^{-2} or J cm^{-2}) taking into account the area of the device

- [ ] Yes
- [x] No

The density values are not important in our experiment, so we did not measure them

6. Alternative explanations

Reasoning as to why alternative explanations have been ruled out as responsible for the emission characteristics
e.g. amplified spontaneous, directional scattering; modification of fluorescence spectrum by the cavity

- [ ] Yes
- [x] No

This information is not necessary and sensitive to our experiment.

7. Theoretical analysis

Theoretical analysis that ensures that the experimental values measured are realistic and reasonable
e.g. laser threshold, linewidth, cavity gain-loss, efficiency

- [x] Yes
- [ ] No

In Supplementary Information Note 3, we measured the laser power and ensure the steady state temperature rise is within 10 K.

8. Statistics

Number of devices fabricated and tested

- [x] Yes
- [ ] No

In Supplementary Information Figure S11, the number of test is shown. In Supplementary Information Figure S3, we tested the beam spatial shape and intensity profile of our laser. We only use one laser device to conduct the experiment

Statistical analysis of the device performance and lifetime (time to failure)

- [x] Yes
- [ ] No

We make sure thermo reflectance signal is stable every time before test, which indicate a laser stability. We did not analyze the lifetime of the device.
